# Temperament, sleep quality, and insomnia severity in university students: Examining the mediating and moderating role of sleep hygiene

**DOI:** 10.1371/journal.pone.0251557

**Published:** 2021-07-15

**Authors:** Angela F. Lukowski, Dmitry Tsukerman

**Affiliations:** Department of Psychological Science, UC Irvine, Irvine, California, United States of America; Charite Medical University Berlin, GERMANY

## Abstract

University students commonly experience sleep problems which have implications for daily functioning and academic achievement. For this reason, research is needed to identify modifiable individual difference variables that may contribute to better sleep in this population. Temperament and sleep hygiene may be two such factors. As part of a larger study, 167 university students (61.7% female) completed online questionnaires that inquired about temperament (the Adult Temperament Questionnaire; ATQ), sleep hygiene behavior (the Sleep Hygiene Index; SHI), global sleep quality (the Pittsburgh Sleep Quality Index; PSQI), and insomnia severity (the Insomnia Severity Index; ISI). Correlations amongst the included measures were in the predicted direction: effortful control was negatively associated with the SHI composite, PSQI global scores, and ISI scores; extraversion was negatively related to PSQI global scores; and negative affect was positively associated with the SHI composite and ISI scores. In addition, the SHI composite mediated the association between effortful control and the PSQI global scores as well as the association between negative affect and PSQI global scores; similar patterns of mediation were found when considering ISI scores, although the direct effects differed. That is, negative affect was directly associated with ISI scores but not PSQI global scores. These findings suggest that interventions designed enhance effortful control, reduce negative affect, and improve sleep hygiene may contribute to better global sleep quality and decrease insomnia in university students.

## Introduction

University students commonly experience sleep problems, including poor global sleep quality and insomnia, with negative consequences for daytime functioning academic achievement. For this reason, research is needed to identify modifiable individual difference variables that may contribute to better sleep in this population. Temperament is one set of individual difference variables that has been associated with sleep in university student samples [1,2]. Given the small number of studies conducted to date, however, as well as the potential implications of improving sleep and daytime functioning in this population, the present research was conducted to (1) examine associations among temperament, sleep hygiene behavior, and insomnia severity in university students as compared to analyses focused on temperament, sleep hygiene behavior, and global sleep quality, to (2) identify the mediating and moderating role of sleep hygiene behavior in explaining the observed relations [1,2], and to (3) replicate previous research documenting correlations between temperament and global sleep quality.

Temperament refers to a constellation of biologically-based individual differences in reactivity and regulation that emerge and develop within the broader cultural context [3]. Whereas reactivity relates to the manner and extent to which one reacts behaviorally and physiologically to environmental stimuli, regulation refers to the ability to alter or modulate those responses [4]. Temperament has been conceptualized as a precursor or contributor to later personality [5], and specific dimensions of adult temperament are significantly associated with particular aspects of personality [4]. One of the most widely used measures of adult temperament is the Adult Temperament Questionnaire, which includes 77 questions that are reduced into 13 scales and four factor scores, including effortful control (e.g., activational, attentional, and inhibitory control), extraversion (e.g., high intensity pleasure, positive affect, sociability), negative affect (e.g., discomfort, fear, frustration, and sadness), and orienting sensitivity (e.g., affective, associative, and neutral sensitivity to environmental stimuli) [6]. Additional information about this measure, including brief definitions of each of the included scales, can be found here.

Although a sizeable literature has documented relations between temperament and sleep in infants and young children [7], limited research has examined whether and to what extent temperament is associated with sleep problems in adults. Such research may be particularly informative when considering university student samples, as previous research has revealed poorer sleep quality in this population relative to community samples [8], with recent estimates indicating that over 60% of students report poor quality sleep [9]. In addition, approximately 25% of students report symptoms associated with a diagnosable sleep disorder [10]. This finding is of particular importance when considering insomnia, as a recent meta-analysis [11] indicated that university students are disproportionately affected by this sleep disorder in particular (approximately 19% prevalence rates) relative to community samples (approximately 7%; although estimates vary significantly in relation to how insomnia is operationalized; see reference [12]). Efforts to remedy these sleep problems are particularly important in university student samples, given documented relations between sleep problems, mental health issues [13–15], and poorer academic achievement [16–18].

Two studies conducted to date have examined associations between sleep and temperament in university students. The first study to our knowledge examined these relations in a sample of university students who reported relatively good sleep habits [1], whereas the second study included university students recruited more broadly [2]. The correlational findings were quite consistent across these two studies, with results indicating that effortful control and negative affect were related to global sleep quality in opposite directions (i.e., greater effortful control was related to better quality sleep, whereas increased negative affect was associated with poorer sleep quality). The findings from the study of students with generally good sleep habits also found that orienting sensitivity was related to poorer sleep quality [1], although this finding was not replicated in the study with students recruited more broadly [2].

In addition to examining associations between temperament and global sleep quality, previous research has also focused on the role of sleep hygiene behavior [2]. Sleep hygiene behavior is evaluated by asking participants about the frequency with which they engage in sleep-promoting versus sleep-hindering activities, such as including taking lengthy daytime naps or playing video games or watching television before bed [19]. University students commonly report engaging in activities that are associated with poor sleep hygiene despite demonstrating adequate sleep hygiene awareness [20]. By extension, poorer sleep hygiene behavior has been associated with reduced GSQ in university students [2,21,22]. Poor sleep hygiene behavior is not likely directly responsible for the onset of significant sleep problems such as insomnia, however, but may be involved in their maintenance. That is, individuals experiencing nighttime sleep problems may engage in activities associated with poorer sleep hygiene behavior when they wake during the night (e.g., watching television or using technology), thereby perpetuating the problem [23].

Previous research focused on sleep, temperament, and sleep hygiene behavior has indicated that sleep hygiene behavior mediates the association between both effortful control and global sleep quality on the one hand, as well as the relation between negative affect and global sleep quality on the other [2]; effortful control was also directly associated with global sleep quality. Evidence of mediation was not found when considering measures of extraversion or orienting sensitivity, and the results of the conducted moderation analyses were not statistically significant. These findings indicate that variability in effortful control and negative affect in particular impact global sleep quality through sleep hygiene behavior. The potential implications of these findings warrant additional research in other samples of students as well as extension to other types of sleep problems, such as insomnia, that may have significant implications for daily functioning and academic achievement in this population.

As mentioned previously, the goals of this study were to (1) examine associations among temperament, sleep hygiene behavior, and insomnia severity in university students as compared to analyses focused on temperament, sleep hygiene behavior, and global sleep quality to (2) identify the mediating and moderating role of sleep hygiene behavior in explaining the observed relations [1,2], and to (3) replicate previous research documenting correlations between temperament and global sleep quality. We predicted that effortful control would be negatively associated with insomnia severity and that negative affect would be positively associated with insomnia severity. When considering the mediation analyses, we anticipated that effortful control would be directly associated with insomnia severity, and that effortful control would also impact insomnia severity through sleep hygiene behavior. We made the same predictions for negative affect, as previous research has indicated that depression in the absence of insomnia is a risk factor for developing insomnia over time [24–26]. We did not expect to find evidence of mediation concerning extraversion or orienting sensitivity; we also anticipated that the moderation models conducted for the four temperament factor scores would yield non-significant results.

When considering global sleep quality, we predicted that the findings obtained in this study would replicate those reported previously [2]. In particular, we anticipated that effortful control would be positively associated with sleep hygiene behavior and global sleep quality, extraversion would be unassociated with sleep hygiene behavior and global sleep quality, negative affect would be negatively related to sleep hygiene behavior and unassociated with global sleep quality, and orienting sensitivity would be unrelated to both sleep hygiene behavior and global sleep quality. When considering the mediation models, we expected that sleep hygiene behaviors would mediate the association between effortful control and global sleep quality as well as associations between negative affect and global sleep quality; we also predicted that effortful control would be directly associated with global sleep quality. We did not expect to find evidence of mediation when considering extraversion or orienting sensitivity, and we predicted that the tested moderation models would be non-significant, as has been reported previously [2].

## Materials and method

### Ethics statement

The completion of this study was approved by the Institutional Review Board at the University of California, Irvine (HS #2018–4872). Students were tested individually and signed informed consent statements indicating their willingness to participate.

### Participants

Students at a large public university in the southwestern United States were recruited to participate. Two groups of students, namely college club athletes (n = 70) and non-club athletes (n = 140), were recruited to fulfill the goals of the second author’s dissertation study [27]. Relative to recreational intramural athletes and elite Division 1 National Collegiate Athletic Association (NCAA) athletes, college club athletes are likely best characterized as moderately competitive: athletes commonly try out to obtain a position on the team, members must routinely attend practices and games, and teams compete nationally and regionally against other universities. College club athletes were recruited by research assistants who attended practice events, explained the details of the study to potential participants, and invited athletes to participate. Participants received $40 in cash in appreciation for their participation.

Non-club athletes were recruited through the university Human Subjects Lab Pool (HSLP) and through announcements made by research assistants in large lecture classes. Because only students enrolled in select psychology courses register to participate in research through the Human Subjects Lab Pool, classroom announcements were also used to diversify the student population in the non-club athlete sample. In appreciation for their involvement, participants recruited through the HSLP received one credit of extra credit that they could apply to an eligible psychology course of their choosing; students recruited through classroom announcements received $10 in cash.

Across the combined sample of club athletes and non-athletes (n = 210), the data from 43 participants (n = 13 athletes) were excluded from analysis for the following reasons: 11 students (n = 2 athletes) took medications that could affect their sleep, 7 students (n = 4 athletes) were being treated for a sleep problem, 11 students (n = 3 athletes) were being treated for a sleep problem and were taking medications that could affect their sleep, 12 students (n = 3 athletes) did not complete the sleep inventories or provided data that precluded their analysis, and 2 participants (n = 1 athlete) were being treated for a sleep problem and provided incomplete or unusable sleep data. As such, the final sample (n = 167) included 57 club athletes and 110 non-club athletes.

#### Demographic information

Participants were asked to provide demographic information including gender, age, race, and ethnicity, among others. Participants were also asked to indicate whether they were taking any medication for mood or sleep problems at the time of the study, as well as whether they had a diagnosed sleep condition. Because of formatting differences for one question on the demographic questionnaire, club athletes entered their age into a text box, whereas non-club athletes selected their age on a multiple choice question with response options ranging from 18 years old to over 25 years old. In order to obtain a continuous age variable for all participants, we computed the mean age of the athletes who were over age 25 at the time of the study (n = 4) and replaced the missing data for the non-athletes (n = 3) with this value (i.e., 26 years old).

#### Global sleep quality

Self-reported global sleep quality was assessed using the Pittsburgh Sleep Quality Index (PSQI) [28]. This questionnaire includes 19 items that inquire about various quantitative (e.g., amount of time spent in bed, amount of time spent asleep) and qualitative (e.g., perceived sleep quality, enthusiasm for completing daytime activities) aspects of sleep as experienced over the previous 30 days. The questions are scored to yield seven component scores (daytime dysfunction, sleep duration, sleep efficiency, sleep latency, subjective sleep quality, use of sleep medication) that are scored from 0 to 3, which higher scores indicating poorer sleep quality. These scores are summed to yield one measure of global sleep quality (subsequently referred to as PSQI global scores). Higher scores indicate poorer quality sleep; scores greater than 5 indicate categorically poor sleep quality. This measure has demonstrated adequate sensitivity and specificity in differentiating between community samples and individuals with suspected or documented sleep problems. Measures of internal consistency were also acceptable in these studies, although more recent research has reported somewhat lower levels of reliability for university student samples (α = .61 in reference [1]; α = .52 in reference [2]; α = .46 in the present research).

#### Insomnia severity

Self-reported symptoms of insomnia and the extent to which they interfered with daily life were assessed using the 7-item Insomnia Severity Index (ISI) [29]. The responses to individual items were scored and summed to yield a composite measure of insomnia severity (subsequently referred to as ISI scores). Higher scores indicate more severe insomnia symptomatology. Categorically, scores ranging from 0–7 indicate no insomnia, scores from 8–14 are mild or subthreshold for clinical relevance, scores from 15–21 indicate moderate clinical insomnia, and scores of 22 or more indicate severe clinical insomnia. This measure has demonstrated adequate convergent validity with other measures assessing insomnia in patient populations as well as acceptable internal consistency (α = .78).

#### Sleep hygiene behavior

Sleep hygiene behavior was assessed using the 13-item Sleep Hygiene Index (SHI) [30]. The items inquire as to the extent to which participants generally or usually engaged in various activities associated with poor sleep hygiene (e.g., napping during the day, exercising or playing video games shortly before bed, sleeping on an uncomfortable pillow or bed). Participants rated how frequently they engaged in these behaviors on a 5-point scale. Responses to the individual items were then summed to yield a composite score of sleep hygiene behaviors (subsequently referred to as the SHI composite). Higher scores indicate poorer sleep hygiene (α = .63).

#### Temperament

Adult temperament was assessed using the short form of the ATQ [6]. Participants completed 77 items that assessed how frequently individuals engaged in various everyday behaviors on a 7-point scale; an option was included as well for participants to indicate that they did not experience the represented situation. Higher scores indicate greater identification with the presented statement. The data were reduced to 13 scales that were further reduced into four factor scores based on previous analyses [6], including effortful control (α = .78), extraversion (α = .73), negative affect (α = .84), and orienting sensitivity (α = .73).

### Procedure

College club athletes were tested during Winter and Spring 2019; due to the goals of the dissertation study for which these data were collected, their first sessions always occurred on Tuesdays. Non-athletes were tested during Fall 2019 and Winter 2020; no constraints were imposed on the day of the week on which testing occurred.

Immediately after providing informed consent, participants reported on demographic information and completed an online questionnaire packet including the PSQI [28], the ISI [29], the SHI [30], and the ATQ [6]. Club athletes completed additional tasks and questionnaires that are beyond the scope of this report.

### Data analysis plan

After identifying whether any of the demographic variables should be included as covariates in the primary analyses of interest, we examined whether the various temperament variables were associated with the SHI composite, PSQI global scores, and ISI scores. Simple mediation models were tested using PROCESS Model 4 as described in reference [31]. Each analysis included a temperament factor score as the predictor (x), the SHI composite as the mediator (m), and either the PSQI global score or the ISI score as the outcome (y). For each analysis, mediation was indicated when k = 10,000 bias-corrected bootstrap confidence intervals did not include zero.

Simple moderation models were tested using PROCESS Model 1 as described in reference [31]. Each analysis included a temperament factor score as the predictor (x), the SHI composite as the moderator (w), and either the PSQI global score or the IS score as the outcome (y). To allow for meaningful interpretation of the resulting coefficients, the temperament factor scores and the SHI composite were mean-centered before analysis.

## Results

### Potential covariates

#### Group differences

Because participants were recruited separately based on their identification as club athletes or non-club athletes, we conducted preliminary analyses to examine whether the groups differed on demographic characteristics, the SHI composite, PSQI global scores, ISI scores, and temperament factor scores. These data are shown in Table 1.

**Table 1:** Examination of group differences for athletes and non-athletes (N = 167).

Measures	Overall	Athletes	Non-Athletes
Demographic information			
Sex (% females)	61.7%	59.6%	62.7%
* Race			
% White	42.2%	58.8%	33.3%
% Asian	50.3%	23.5%	64.6%
% other races	7.5%	17.6%	2.1%
* Ethnicity (% Hispanic)	28.1%	33.3%	25.5%
Age (years)	20.35 ±.13	20.30 ±.22	20.37 ±.16
Sleep data			
SHI composite	20.60 ±.46	20.49 ±.80	20.66 ±.57
PSQI global sleep quality	5.67 ±.17	5.51 ±.29	5.76 ±.21
Categorically poor sleep quality	50.9%	45.6%	53.6%
ISI scores	7.14 ±.32	7.12 ±.55	7.15 ±.40
No clinically significant insomnia	60.5%	61.4%	60.0%
Subthreshold insomnia	34.1%	33.3%	34.5%
Moderate clinical insomnia	5.4%	5.3%	5.5%
Temperament factor scores			
Effortful control	4.27 ±.06	4.24 ±.10	4.28 ±.08
* Extraversion	4.48 ±.06	4.73 ±.10	4.36 ±.07
* Negative affect	4.05 ±.06	3.74 ±.10	4.22 ±.07
Orienting sensitivity	4.61 ±.06	4.65 ±.11	4.59 ±.08

Note: None of the study participants reported severe clinical insomnia. Significant and marginal findings are indicated:

* *p* < .05 and ° *p* < .10.

#### Examination of other possible covariates

Correlations were also conducted to identify whether the SHI composite, PSQI global scores, ISI scores, and temperament factor scores were associated with participant age (coded continuously from 18 to 26 years), sex (0 = male or 1 = female), and ethnicity (0 = non-Hispanic or 1 = Hispanic). These data are shown in Table 2. Because participant sex and ethnicity were dichotomously coded for analysis through correlation, we conducted follow-up analyses of variance (ANOVAs) to confirm their statistical significance. The same pattern of findings was maintained across the correlations and ANOVAs. Additional information on these results can be obtained from the corresponding author.

**Table 2:** Correlations conducted to identify potential covariates (N = 167).

Questionnaire measures	Age	Sex	Ethnicity
SHI composite	-.05	.14°	-.10
PSQI global scores	.15°	-.05	-.06
ISI scores	.15°	-.02	-.18*
Temperament factor scores			
Effortful control	.11	-.02	.09
Extraversion	.09	-.17*	-.21*
Negative affect	-.09	.29*	.10
Orienting sensitivity	.09	.18*	.00

Note: The data were coded as follows: Participant age (coded continuously from 18 to 26 years), participant sex (0 = male or 1 = female), and ethnicity (0 = Hispanic or 1 = non-Hispanic). Significant and marginal findings are indicated:

* *p* < .05 and

° *p* < .10.

Because the percentage of Asian students in this sample exceeded the national average at institutions of higher learning across the United States [32], we also conducted analyses to identify whether participant race was associated with any of the collected data (see also reference [2]). In particular, we grouped participants based on whether they identified as Asian, White, or another race. Participant race was unassociated with the sleep data, including the SHI composite, PSQI global scores, and ISI scores. When considering temperament factor scores, participant race was only associated with extraversion: *F*(1, 144) = 3.53, *p* = .03, such that White participants (4.59 ±.10) were more extraverted relative to Asian participants (4.28 ±.09); the scores for White and Asian participants did not differ from those for participants of other races (4.68 ±.23). Participant race was unrelated to effortful control, negative affect, and orienting sensitivity.

#### Specification of included covariates

Given the significant differences obtained between athletes and non-athletes, group is included as a categorical covariate in the following analyses. In addition, participant age, sex, and ethnicity were included as demographic covariates given the significant and marginal associations between these variables and the measures of interest. Finally, two dummy-coded variables were created to code for the three levels of race described previously (White, Asian, and other races) given the differential distribution of races by group. These same variables were included as covariates in previous research [2] examining associations between the SHI composite, temperament, and PSQI global scores in undergraduate students.

### Partial correlations

Partial correlations among the various measures of interest (temperament factor scores and scales with measures of sleep, including the SHI composite, PSQI global score, and ISI scores) are shown in Table 3.

**Table 3:** Partial correlations between sleep variables and temperament factors and scales.

Temperament	SHI composite	PSQI global score	ISI score
Effortful control	-.26*	-.32*	-.37*
Activation control	-.21*	-.23*	-.22*
Attentional control	-.28*	-.30*	-.37*
Inhibitory control	-.10	-.23*	-.23*
Extraversion	-.08	-.25*	-.12
High intensity pleasure	.10	-.07	.01
Positive affect	-.09	-.28*	-.23*
Sociability	-.16	-.18*	-.04
Negative affect	.23*	.14	.24*
Discomfort	.07	.09	.14
Fear	.20*	.11	.16
Frustration	.17	.11	.21*
Sadness	.25*	.10	.20*
Orienting sensitivity	.05	-.06	.05
Affective perceptual sensitivity	-.07	.02	.10
Associative sensitivity	.29*	.01	.08
Neutral perceptual sensitivity	-.10	-.18*	-.06

Note: Correlations were conducted controlling for group (0 = athletes or 1 = non-athletes), participant sex (0 = male or 1 = female), participant age (coded continuously from 18 to 26 years), race (with two dummy-coded variables indicating White, Asian, or other races), and ethnicity (0 = Hispanic or 1 = non-Hispanic). Significant findings are indicated:

* *p* < .05.

### Mediation analyses

#### Effortful control (Fig 1A)

**Fig 1:**
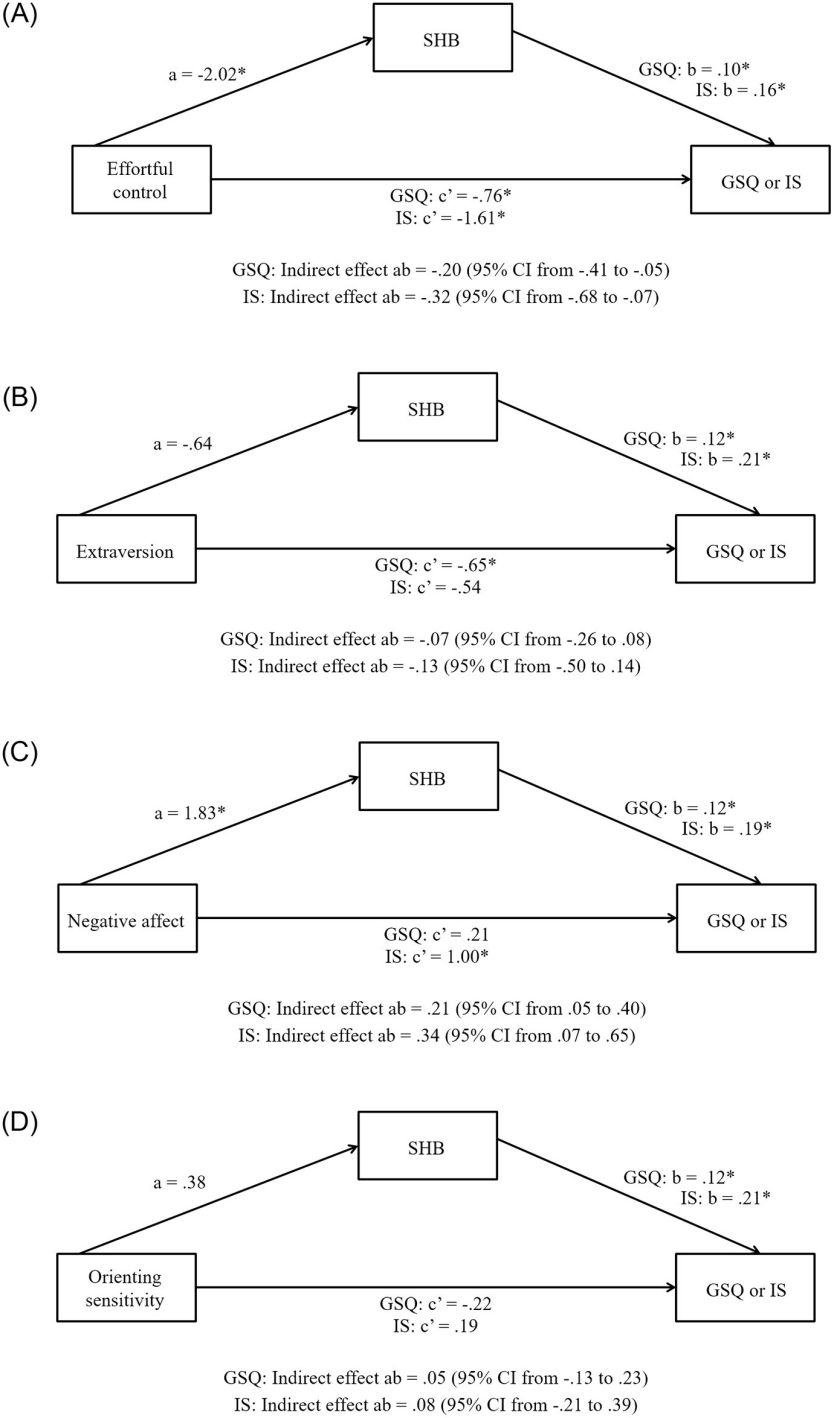
Panel A. Simple mediation analyses: Effortful control. Simple mediation analyses were conducted to identify whether the SHI composite mediated associations between (1) effortful control and PSQI global scores as well as (2) effortful control and ISI scores. Covariates were included in the models as described in the Results section. Significant effects are indicated: * *p* < .05. Panel B. Simple mediation analyses: Extraversion. Simple mediation analyses were conducted to identify whether the SHI composite mediated associations between (1) extraversion and PSQI global scores and (2) extraversion and ISI scores. Covariates were included in the models as described in the Results section. Significant effects are indicated: * *p* < .05. Panel C. Simple mediation analyses: Negative affect. Simple mediation analyses were conducted to identify whether the SHI composite mediated associations between (1) negative affect and PSQI global scores and (2) negative affect and ISI scores. Covariates were included in the models as described in the Results section. Significant effects are indicated: * *p* < .05. Panel D. Simple mediation analyses: Orienting sensitivity. Simple mediation analyses were conducted to identify whether the SHI composite mediated associations between (1) orienting sensitivity and PSQI global scores and (2) orienting sensitivity and ISI scores. Covariates were included in the models as described in the Results section. Significant effects are indicated: * *p* < .05.

Effortful control was negatively associated with the SHI composite in both tested models, and the SHI composite was negatively related to PSQI global scores and ISI scores. In addition, the direct effects of effortful control on PSQI global scores and ISI scores were statistically reliable, as were the indirect effects of effortful control on PSQI global scores and ISI scores through the SHI composite.

#### Extraversion (Fig 1B)

Extraversion was not associated with the SHI composite in either tested model, whereas the SHI composite was positively related to PSQI global scores and ISI scores. The direct effect of extraversion on PSQI global scores was statistically reliable, whereas the direct effect of extraversion on ISI scores was not. In both models, the indirect effects of extraversion on PSQI global scores and ISI scores through the SHI composite were not statistically significant.

#### Negative affect (Fig 1C)

Negative affect was positively associated with the SHI composite in both tested models, and the SHI composite was positively related to PSQI global scores and ISI scores. The direct effect of negative affect on PSQI global scores was not statistically significant, whereas the direct effect of negative affect on ISI scores was statistically reliable. In addition, the indirect effects of negative affect on PSQI global scores and ISI scores through the SHI composite were significant.

#### Orienting sensitivity (Fig 1D)

Orienting sensitivity was not associated with the SHI composite in either tested model, although the SHI composite was positively related to PSQI global scores and ISI scores. The direct effects of orienting sensitivity on PSQI global scores and ISI scores were not statistically significant, nor were the indirect effects of orienting sensitivity on PSQI global scores or ISI scores through the SHI composite.

### Moderation analyses

#### Effortful control (Fig 2A)

**Fig 2:**
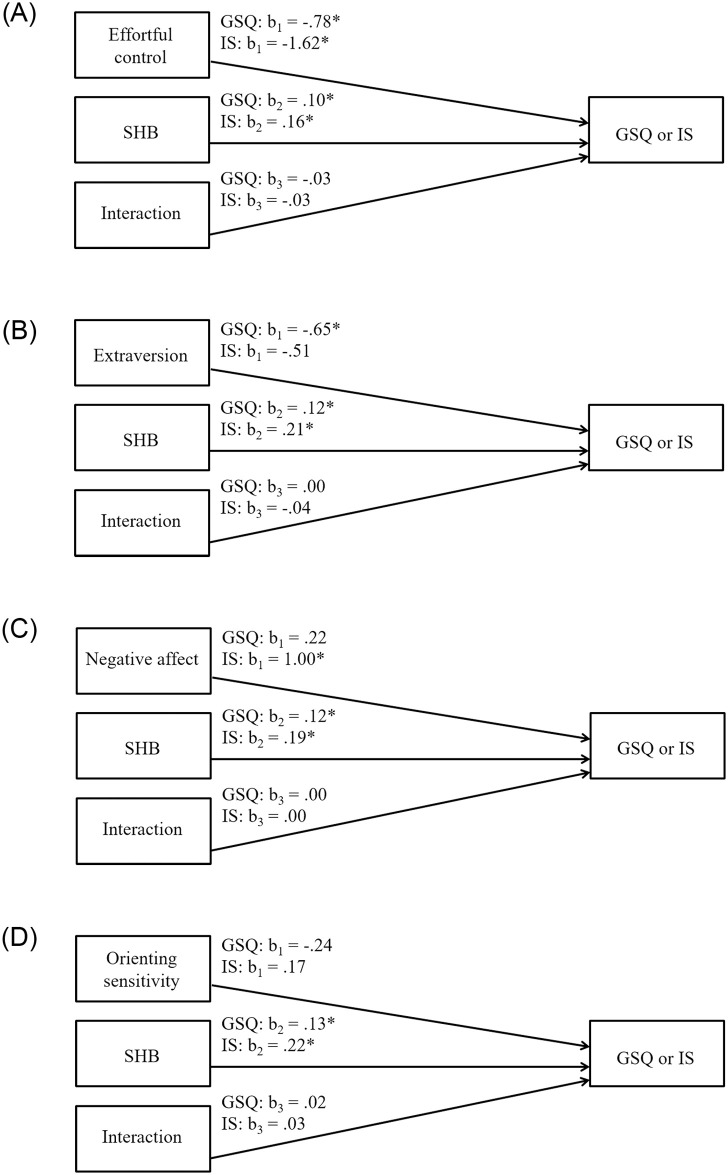
Panel A. Simple moderation analyses: Effortful control. Simple moderation analyses were conducted to identify whether the SHI composite moderated the effect of (1) effortful control on PSQI global scores and (2) effortful control on ISI scores. Covariates were included in the models as described in the Results section. Significant effects are indicated: * *p* < .05. Panel B. Simple moderation analyses: Extraversion. Simple moderation analyses were conducted to identify whether the SHI composite moderated the effect of (1) extraversion on PSQI global scores and (2) extraversion on ISI scores. Covariates were included in the models as described in the Results section. Significant effects are indicated: * *p* < .05. Panel C. Simple moderation analyses: Negative affect. Simple moderation analyses were conducted to identify whether the SHI composite moderated the effect of (1) negative affect on PSQI global scores and (2) negative affect on ISI scores. Covariates were included in the models as described in the Results section. Significant effects are indicated: * *p* < .05. Panel D. Simple moderation analyses: Orienting sensitivity. Simple moderation analyses were conducted to identify whether the SHI composite moderated the effect of (1) orienting sensitivity on PSQI global scores and (2) orienting sensitivity on ISI scores. Covariates were included in the models as described in the Results section. Significant effects are indicated: * *p* < .05.

Effortful control was negatively associated with PSQI global scores and ISI scores, whereas the SHI composite was positively related to PSQI global scores and ISI scores. The interaction between effortful control and the SHI composite did not significantly predict either PSQI global scores or ISI scores.

#### Extraversion (Fig 2B)

Extraversion was negatively associated with PSQI global scores but was unrelated to ISI scores; the SHI composite was positively related to both PSQI global scores and ISI scores. The interaction between extraversion and the SHI composite did not significantly predict either PSQI global scores or ISI scores.

#### Negative affect (Fig 2C)

Negative affect was not related to PSQI global scores but was significantly associated with ISI scores; the SHI composite score was positively related to both PSQI global scores and ISI scores. The interaction between negative affect and the SHI composite did not predict either PSQI global scores or ISI scores.

#### Orienting sensitivity (Fig 2D)

Orienting sensitivity was not related to PSQI global scores or ISI scores, whereas the SHI composite was positively associated with PSQI global scores and ISI scores. The interaction between orienting sensitivity and the SHI composite did not predict either PSQI global scores or ISI scores.

## Discussion

The present study was conducted to (1) examine associations among temperament, sleep hygiene behaviors, and insomnia severity in university students as compared to analyses focused on temperament, sleep hygiene behavior, and global sleep quality, to (2) identify the mediating and moderating role of sleep hygiene behavior in explaining the observed relations [1,2], and to (3) replicate previous research documenting correlations between temperament and GSQ. In brief, (1) similar patterns of correlations were obtained across sleep measures for effortful control, with divergent findings obtained for negative affect, extraversion, and orienting sensitivity. Additional analyses (2) indicated that sleep hygiene behavior mediated associations between effortful control and both sleep measures, with a similar pattern of mediation obtained for negative affect; evidence of mediation was not found for extraversion or orienting sensitivity, and none of the tested moderation models were statistically significant. In addition, and as described below, (3) the findings presented herein largely replicate previous research documenting associations between temperament and global sleep quality in university students [1,2].

The findings from the correlational analyses indicated that increased effortful control was related to less severe insomnia and better global sleep quality; increased effortful control was also associated with better sleep hygiene. The consistency of the findings across sleep measures may be due to the mediating effect of sleep hygiene on ISI scores and PSQI global scores. That is, sleep hygiene mediated the association between effortful control and sleep for both of these dependent measures. This result, obtained through self-report in this study, is consistent with previous behavioral research indicating that better inhibitory control, a sub-component of effortful control, is positively related to sleep hygiene behavior [33,34]; other work has established that good sleep hygiene is associated with better quality sleep [2,21,22] and reduced insomnia [23]. Given these behavioral findings, it is somewhat unclear why the correlation between inhibiotory control and the SHI composite was not statistically significant in this study, although at least one previous study has revealed lack of consistency between behavioral and personality-based self-report measures of impulsivity [35]. Nevertheless, these findings suggest that future experimental work should be conducted to identify whether training in aspects of effortful control improves sleep problems in unversity students, either directly or through sleep hygiene behaviors.

Despite the apparent consistency in findings for effortful control across sleep measures, a different pattern of correlations was obtained for insomnia and global sleep quality when considering negative affect. Greater insomnia severity was related to increased negative affect, frustration, and sadness, whereas significant associations were not found when considering global sleep quality. These distinct associations are substantiated in previous research. In one study, for example, undergraduate students with insomnia reported greater depressive symptoms relative to students with behaviorally induced insufficient sleep syndrome (BIISS; [36]), a condition in which individuals choose to restrict their time in bed (frequently to engage in social activities), ultimately leading to excessive daytime sleepiness. In addition, participants with insomnia and those with BIISS reported greater symptoms of depression relative to individuals with neither disorder. Previous research indicates that insomnia is both a cause and consequence of depression [24–26], and depression has been widely associated with poorer academic achievement in university students [37,38]. For these reasons, staff at university mental health and wellness centers should work to identify students with specific sleep problems (including insomnia, BIISS, and poor quality sleep) and make targeted recommendations to improve their sleep with the goal of enhancing daily functioning and academic performance.

An additional pathway to improved sleep in individuals higher in negative affect may be through improved sleep hygiene behaviors. As with effortful control, the results of the conducted mediation models indicated that participants with greater negative affect had poorer sleep hygiene, and sleep hygiene mediated the association between negative affect and both insomnia severity and global sleep quality. In addition, previous research has indicated that poorer attentional control, an aspect of effortful control, interacted with negative emotionality to predict insomnia in young adults [39]. Previous work has shown the benefits of sleep hygiene training in improving sleep and cognitive functioning in university students (for a review, see reference [18]). As such, interventions aimed at improving sleep hygiene may also have measurable effects on global sleep quality, insomnia, and academic achievement in university student samples.

Future research examining the unique sleep problems experienced by undergraduate students may also inform the unique associations between PSQI global sleep quality and measures of extraversion obtained in this research. Contrary to the findings presented herein, previous research has indicated that greater sociability is associated with sleep problems in adolescents [40] and university students [41, when using a personality-based measure of extraversion]. These findings seem intuitive, as adolescents and young adults who endorse greater levels of sociability might spend more time engaging with friends into the evening hours. Somewhat surprisingly, the opposite association was observed in this study, as greater extraversion and sociability were related to better global sleep quality. We suggest that this unexpected and counterintuitive finding may have resulted from the targeted inclusion of club athletes as participants, as these students reported greater extraversion relative to non-club athletes. Greater levels of extraversion in athlete relative to non-athlete samples have been reported in other work as well [42,43], along with evidence of increased well-being in student athletes compared to non-athletes [42]. As such, higher levels of self-reported extraversion in college club athletes may not be associated with late-night socialization with friends, but may instead be associated with “third variables” that have been related to better nighttime sleep. For example, college club athletes may maintain a broader social support network of teammates and friends than other students, and more supportive friendship networks have been associated with better nighttime sleep quality [44]; the regular exercise habits experienced by college club athletes may also contribute to better quality nighttime sleep [45,46]. These findings suggest that college club athletes may report lower levels of BIISS relative to adolescents with higher levels of extraversion, although this possibility has not yet been empirically evaluated to our knowledge. Given these findings, future research on sleep and temperament would be best focused on experimental work, with the ultimate goal of establishing causal relations. In one relevant study, undergraduate students could be randomized to participate in inhibitory control training (see reference [47]) and informational sessions about sleep hygiene behavior (see reference [48]); other participants would be assigned to control conditions, resulting in a 2 (inhibitory control: training or control) x 2 (sleep hygiene: training or control) experimental design. If the experimental manipulation is effective, students who participate in both training conditions should demonstrate improved sleep hygiene behaviors and better sleep outcomes (i.e., better global sleep quality and reduced insomnia) from pre- to post-test relative to students in the other conditions; controlling for a priori sleep hygiene behaviors, students who participate in both control conditions should report the poorest sleep hygiene and sleep outcomes. The impact of individual difference variables will be most evident for students who participate in one experimental group and one control group. That is, students who receive inhibitory control training in the absence of sleep hygiene training will likely experience better sleep hygiene and sleep outcomes to the extent that they are aware of good sleep hygiene practices at baseline. Students who receive sleep hygiene but not inhibitory control training may report better sleep hygiene and sleep outcomes if they have higher endogenous levels of inhibitory control [47]; it is not anticipated that better sleep hygiene and sleep outcomes will result for students who do not have the cognitive control to implement sleep hygiene behaviors successfully. Ultimately, the results of this and other experimental research will serve as the foundation for interventions designed to positively impact students’ sleep quality, daily functioning, and academic achievement.

Future research should also be conducted to account for some of the limitations of the present study. One limitation of the conducted research is that the reported associations focus only on subjective reports of sleep without objective verification. Future research should identify whether the same pattern of findings emerges when subjective reports and objective measures are used. Indeed, previous reports have revealed non-significant associations between actigraphy, polysomnography, and self-report [49], along with significant relations between self-reported sleep quality and indices of psychological well-being [49,50]. Identifying whether the findings reported in this work are maintained when considering objective measures has important implications, as future researchers should attempt to improve characteristics of sleep that are most strongly associated with daytime functioning and academic achievement in university students.

In sum, identifying modifiable individual difference factors that contribute to better sleep in university students is important because undergraduate student sleep problems have been extensively associated with worse academic achievement. A recent review article [18] documents associations between poorer sleep and impaired procedural and declarative memory, worse performance on prefrontal-based measures of executive functioning, and reduced academic achievement. Sleep loss has been associated with impaired cognitive functioning, whereas experimental interventions designed to increase the duration of nighttime sleep result in improved performance. Although it is unknown whether interventions designed enhance effortful control, reduce negative affect, and improve sleep hygiene may contribute to greater nighttime sleep duration in university students, increasing the amount of time slept at night may be a low-cost and effective means of improving learning and academic performance in this population. Future research must be conducted to identify whether different interventions are needed for individuals who have BIISS, insomnia, or poor global sleep quality, but the potential benefits of improving sleep in university students are significant, both when considering implications for individual students and society at large.

## Conclusions

The conducted research revealed distinct correlations between temperament and global sleep quality, on the one hand, and temperament and insomnia severity on the other when considering negative affect, extraversion, and orienting sensitivity; comparable effects were found when considering effortful control. Sleep hygiene mediated associations between effortful control and both sleep measures; analogous findings were observed for negative affect. Distinct direct effects were observed for negative affect, however, such that negative affect was directly associated with insomnia severity but not global sleep quality. These findings confirm previous research indicating greater associations between insomnia and depression relative to other sleep problems [36]. Taken together, our results suggest that interventions designed enhance effortful control, reduce negative affect, and improve sleep hygiene may contribute to better global sleep quality and decrease insomnia in university students. Future experimental research should be conducted to examine these possibilities, as the potential benefits of improving sleep in university students are great.

## References

[1] Lukowski AF, Milojevich HM. Sleep quality and temperament among university students: Differential associations with nighttime sleep duration and sleep disruptions. Behav Sleep Med. 2015;13: 217–230. doi: 10.1080/15402002.2013.855214 24611534

[2] Lukowski AF, Eales L, Tsukerman D. Sleep hygiene mediates, but does not moderate, associations between temperament and sleep quality in university students. Behav Med. 2018;45: 282–293. doi: 10.1080/08964289.2018.1509052 30481141

[3] Rothbart MK, Derryberry D. Development of individual differences in temperament. In: Lamb ME, Brown AL, editors. Advances in developmental psychology. Vol. 1. Hillsdale: Erlbaum; 1981. p. 37–86.

[4] Rothbart MK, Ahadi SA, Evans DE. Temperament and personality: Origins and outcomes. J Pers Soc Psychol. 2000;78: 122–135. doi: 10.1037//0022-3514.78.1.122 10653510

[5] Cloninger CR. Temperament and personality. Curr Opin Neurobiol. 1994;4: 266–273. doi: 10.1016/0959-4388(94)90083-3 8038587

[6] Evans DE, Rothbart MK. Developing a model for adult temperament. J Res Pers. 2007;41: 868–888.

[7] Spruyt K, Aitken RJ, So K, Charlton M, Adamson TM, Horne RSC. Relationship between sleep/wake patterns, temperament and overall development in term infants over the first year of life. Early Hum Dev. 2008;84: 289–296. doi: 10.1016/j.earlhumdev.2007.07.002 17707119

[8] Buboltz WC, Brown F, Soper B. Sleep habits and patterns of college students: A preliminary study. J Am Coll Heal. 2001;50: 131–135. doi: 10.1080/07448480109596017 11765249

[9] Lund HG, Reider BD, Whiting AB, Prichard JR. Sleep patterns and predictors of disturbed sleep in a large population of college students. J Adolesc Heal. 2010;46: 124–132. 10.1016/j.jadohealth.2009.06.016 20113918

[10] Gaultney JF. The prevalence of sleep disorders in college students: Impact on academic performance. J Am Coll Heal. 2010;59: 91–97. doi: 10.1080/07448481.2010.483708 20864434

[11] Jiang XL, Zheng XY, Yang J, Ye CP, Chen YY, Zhang ZG, et al. A systematic review of studies on the prevalence of insomnia in university students. Public Health. 2015;129: 1579–1584. doi: 10.1016/j.puhe.2015.07.030 26298588

[12] Ohayon MM. Epidemiology of insomnia: What we know and what we still need to learn. Sleep Med Rev. 2002;6: 97–111. doi: 10.1053/smrv.2002.0186 12531146

[13] Gress-Smith JL, Roubinov DS, Andreotti C, Compas BE, Luecken LJ. Prevalence, severity and risk factors for depressive symptoms and insomnia in college undergraduates. Stress Heal. 2015;31: 63–70. doi: 10.1002/smi.2509 23897800

[14] Milojevich HM, Lukowski AF. Sleep and mental health in undergraduate students with generally healthy sleep habits. PLoS One. 2016;11: 1–14. doi: 10.1371/journal.pone.0156372 27280714 PMC4900547

[15] Ustinov Y, Lichstein KL, Vander Wal GS, Taylor DJ, Riedel BW, Bush AJ. Association between report of insomnia and daytime functioning. Sleep Med. 2010;11(1): 65–68. doi: 10.1016/j.sleep.2009.07.009 19783473

[16] Gilbert SP, Weaver CC. Sleep quality and academic performance in university students: A wake-up call for college psychologists. J College Stud Psychother. 2010;24: 295–306.

[17] Trockel MT, Barnes MD, Egget DL. Health-related variables and academic performance among first-year college students: Implications for sleep and other behaviors. J Am Coll Heal. 2000;49:125–131. doi: 10.1080/07448480009596294 11125640

[18] Curcio G, Ferrara M, De Gennaro L. Sleep loss, learning capacity and academic performance. Sleep Med Rev. 2006;10: 323–337. doi: 10.1016/j.smrv.2005.11.001 16564189

[19] Mastin DF, Bryson J, Corwyn R. Assessment of sleep hygiene using the Sleep Hygiene Index. J Behav Med. 2006;29: 223–227. doi: 10.1007/s10865-006-9047-6 16557353

[20] Felix VA, Campsen NA, White A, Buboltz WC. College students’ prevalence of sleep hygiene awareness and practices. Adv Soc Sci Res J. 2017;4: 91–105.

[21] Kang J-H, Chen S-C. Effects of an irregular bedtime schedule on sleep quality, daytime sleepiness, and fatigue among university students in Taiwan. BMC Public Health. 2009;9: 248. doi: 10.1186/1471-2458-9-248 19615098 PMC2718885

[22] Suen LKP, Tam WWS, Hon KL. Association of sleep hygiene-related factors and sleep quality among university students in Hong. Hong Kong Med J. 2010;16: 180–185. 20519753

[23] Stepanski EJ, Wyatt JK. Use of sleep hygiene in the treatment of insomnia. Sleep Med Rev. 2003;7: 215–225. doi: 10.1053/smrv.2001.0246 12927121

[24] Sivertsen B, Salo P, Mykletun A, Hysing M, Pallesen S, Krokstad S, et al. The bidirectional association between depression and insomnia: The HUNT study. Psychosom Med. 2012;74: 758–765. doi: 10.1097/PSY.0b013e3182648619 22879427

[25] Jannsoon-Fröjmark M, Lindblom K. A bidirectional relationship between anxiety and depression, and insomnia? A prospective study in the general population. J Psychosom Res. 64: 443–449. doi: 10.1016/j.jpsychores.2007.10.016 18374745

[26] Lustberg L, Reynolds CF. 2000. Depression and insomnia: Questions of cause and effect. Sleep Med Rev. 2000;4: 253–262. doi: 10.1053/smrv.1999.0075 12531168

[27] Tsukerman D. Sleep in college club athletes: Patterns, correlates, and comparisons with college non-athletes [dissertation]. University of California, Irvine; 2020.

[28] Buysse DJ, Reynolds CF, Monk TF, Berman SR, Kupfer DJ. The Pittsburgh Sleep Quality Index: A new instrument for psychiatric practice and research. Psychiatry Res. 1989;28: 193–213. doi: 10.1016/0165-1781(89)90047-4 2748771

[29] Bastien CH, Vallières A, Morin CM. Validation of the insomnia severity index as an outcome measure for insomnia research. Sleep Med. 2001;2: 297–307. doi: 10.1016/s1389-9457(00)00065-4 11438246

[30] Mastin DF, Bryson J, Corwyn R. Assessment of sleep hygiene using the Sleep Hygiene Index. J Behav Med. 2006;29: 223–227. doi: 10.1007/s10865-006-9047-6 16557353

[31] Hayes AF. Introduction to mediation, moderation, and conditional process analysis: A regression-based approach. 2nd ed. New York: The Guilford Press; 2017.

[32] National Center for Education Statistics. Total fall enrollment in degree-granting postsecondary institutions, by level of enrollment, sex, attendance status, and race/ethnicity or nonresident alien status of student: Selected years, 1976 through 2018. 2019. https://nces.ed.gov/programs/digest/d19/tables/dt19_306.10.asp.

[33] Hall PA, Elias LJ, Crossley M. Neurocognitive influences on health behavior in a community sample. Health Psychol. 2006;6: 778–782. 10.1037/0278-6133.25.6.778 17100506

[34] Kor K., Mullan BA. Sleep hygiene behaviours: An application of the theory of planned behaviour and the investigation of perceived autonomy support, past behavior and response inhibition. Psychol Health. 2011;26: 1208–1224. doi: 10.1080/08870446.2010.551210 21678170

[35] Reynolds B, Ortengren A, Richards JB, de Wit H. Dimensions of impulsive behavior: Personality and behavioral measures. Pers Individ Dif. 2006;40: 305–315.

[36] Williams AB, Dzierzewski JM, Griffin SC, Lind MJ, Dick D, Rybarczyk BD. Insomnia disorder and behaviorally induced insufficient sleep syndrome: Prevalence and relationship to depression in college students. Behav Sleep Med. 2020;18: 275–286. doi: 10.1080/15402002.2019.1578772 30789063 PMC6814500

[37] DeRoma VM, Leach JB, Leverett JP. The relationship between depression and college academic performance. Coll Stud J. 2009;43: 325–334.

[38] Hysenbegasi A, Hass SL, Rowland CR. The impact of depression on the academic productivity of university students. J Ment Health Policy Econ. 2005;8: 145–151. 16278502

[39] Mitchell L, Mogg K, Bradley BP. Relationships between insomnia, negative emotionality and attention control. Sleep Biol Rhythms. 2012;10: 237–243.

[40] Moore M, Slane J, Mindell JA, Burt SA, Klump KL. Sleep problems and temperament in adolescents. Child Care Health Dev. 2011;37: 559–562. doi: 10.1111/j.1365-2214.2010.01157.x 21083682 PMC3110528

[41] Raynor DA, Levine H. Associations between the five-factor model of personality and health behaviors among college students. J Am Coll Heal. 2009;58: 73–82. doi: 10.3200/JACH.58.1.73-82 19592356

[42] Aries E, McCarthy D, Salovey P, Banaji MR. A comparison of athletes and non-athletes at highly selective colleges: Academic performance and personal development. Res High Educ. 2004;45: 577–602.

[43] Eagleton JR, McKelvie SJ, de Man A. Extraversion and neuroticism in team sport participants, individual sport participants, and nonparticipants. 2007;105: 265–75. 10.2466/pms.105.1.265-275 17918575

[44] Nordin M, Westerholm P, Alfredsson L, Akerstedt T. Social support and sleep: Longitudinal relationships from the WOLF study. Psychology. 2012;3: 1223–1230.

[45] Kredlow MA, Capozzoli MC, Hearon BA, Calkins AW, Otto MW. The effects of physical activity on sleep: A meta-analytic review. J Behav Med. 2015;38: 427–449. doi: 10.1007/s10865-015-9617-6 25596964

[46] Wang F, Boros S. The effect of physical activity on sleep quality: A systematic review. Eur J Physiother. 2019: 1–8.

[47] Todd J, Mullan. The role of self-regulation in predicting sleep hygiene in university students. Psychol Health Med. 2013;18: 275–288. doi: 10.1080/13548506.2012.701756 22788412

[48] Brown FC, Buboltz WC Jr., Soper B. Development and evaluation of the Sleep Treatment and Education Program for Students (STEPS). J Am Coll Heal. 2006;54: 231–237. 10.3200/JACH.54.4.231-237 16450848

[49] Grandner MA, Kripke DF, Yoon I-Y, Youngstedt SD. Criterion validity of the Pittsburgh Sleep Quality Index: Investigation in a non-clinical sample. Sleep Biol Rhythms. 2006;4: 129–139. doi: 10.1111/j.1479-8425.2006.00207.x 22822303 PMC3399671

[50] Gregory AM, Buysse DJ, Willis TA, Rijsdijk F V., Maughan B, Rowe R, et al. Associations between sleep quality and anxiety and depression symptoms in a sample of young adult twins and siblings. J Psychosom Res. 2011;71: 250–255. doi: 10.1016/j.jpsychores.2011.03.011 21911103

